# Speech-Gesture Matching and Schizotypal Traits: A Network Approach

**DOI:** 10.1093/schbul/sbae134

**Published:** 2024-07-24

**Authors:** Bertalan Polner, Hamidreza Jamalabadi, Bianca M van Kemenade, Jutta Billino, Tilo Kircher, Benjamin Straube

**Affiliations:** Institute of Psychology, ELTE, Eötvös Loránd University, Budapest, Hungary; Donders Institute for Brain, Cognition and Behaviour, Radboud University, Nijmegen, The Netherlands; Department of Psychiatry and Psychotherapy, University of Marburg, Marburg, Germany; Center for Psychiatry, Justus Liebig University Giessen, Giessen, Germany; Center for Mind, Brain, and Behavior (CMBB), University of Marburg, Marburg, Germany, and Justus Liebig University Giessen, Giessen, Germany; Center for Mind, Brain, and Behavior (CMBB), University of Marburg, Marburg, Germany, and Justus Liebig University Giessen, Giessen, Germany; Experimental Psychology, Lifespan Neuropsychology, Justus Liebig University Giessen, Giessen, Germany; Department of Psychiatry and Psychotherapy, University of Marburg, Marburg, Germany; Center for Mind, Brain, and Behavior (CMBB), University of Marburg, Marburg, Germany, and Justus Liebig University Giessen, Giessen, Germany; Department of Psychiatry and Psychotherapy, University of Marburg, Marburg, Germany; Center for Mind, Brain, and Behavior (CMBB), University of Marburg, Marburg, Germany, and Justus Liebig University Giessen, Giessen, Germany

**Keywords:** psychosis spectrum, social cognition, interpersonal communication, network analysis, nonverbal communication

## Abstract

**Background and Hypothesis:**

Impaired speech-gesture matching has repeatedly been shown in patients with schizophrenia spectrum disorders. Here, we tested the hypothesis that schizotypal traits in the general population are related to reduced speech-gesture matching performance and reduced self-reports about gesture perception. We further explored the relationships between facets of schizotypy and gesture processing in a network model.

**Study Design:**

Participants (1094 mainly healthy adults) were presented with concrete or abstract sentences accompanied with videos showing related or unrelated gestures. For each video, participants evaluated the alignment between speech and gesture. They also completed self-rating scales about the perception and production of gestures (Brief Assessment of Gesture scale) and schizotypal traits (Schizotypal Personality Questionnaire—Brief 22-item version). We analyzed bivariate associations and estimated a non-regularized partial Spearman correlation network. We characterized the network by analyzing bridge centrality and controllability metrics of nodes.

**Study Results:**

We found a negative relationship between both concrete and abstract gesture-speech matching performance and overall schizotypy. In the network, disorganization had the highest average controllability and it was negatively related to abstract speech-gesture matching. Bridge centralities indicated that self-reported production of gestures to enhance communication in social interactions connects self-reported gesture perception, schizotypal traits, and gesture processing task performance.

**Conclusion:**

The association between impaired abstract speech-gesture matching and disorganization supports a continuum between schizophrenia and schizotypy. Using gestures to facilitate communication connects subjective and objective aspects of gesture processing and schizotypal traits. Future interventional studies in patients should test the potential causal pathways implied by this network model.

## Introduction

Gestures support communication by emphasizing the content of a verbal message.^1^ Deficits in the perception and production of gestures are apparent in patients with schizophrenia and individuals at high risk for psychosis,^2–10^ and matching speech with gestures seems to be especially impaired in patients.^5,11,12^ However, it remains unknown whether impaired speech-gesture matching is related to schizotypy in the general population.

Schizotypy is a set of personality traits that are continuous indicators of schizophrenia risk and provide insight into its etiology.^13^ High levels of schizotypy predict elevated risk of future schizophrenia,^14^ and accumulating evidence shows an overlap between schizotypy and schizophrenia in terms of phenomenology, cognition, and some structural and functional brain alterations.^15–17^ Thus, one may expect subtle impairments of gesture processing in individuals with high schizotypy.

From a theoretical perspective, these associations can emerge from complex interactions. Throughout development, impaired social cognition (including gesture processing) can make it difficult and stressful to be with others, and such experiences may culminate in avoiding social contact and increase the risk of victimization,^18,19^ thereby triggering vicious loops promoting the development of negative and positive schizotypy, respectively. Considering such reciprocal interactions further motivates the abovementioned continuum approach: in patients, social cognitive impairments might be secondary to the disease itself. We argue that such confounding is less likely to occur in association with schizotypy in the general population.

Empirically, the specific associations between dimensions of schizotypy and social cognition have been ambiguous. Impaired context integration is associated with thought disorder traits,^20^ which may undermine the processing of social signals. Inferring emotions from gait patterns are related to positive schizotypy and autistic traits,^21^ and facial affect recognition is predicted by interpersonal schizotypy^22,23^ and social anxiety.^22^ Furthermore, neural correlates of mentalizing are related to interpersonal and disorganized schizotypy.^24^ Regarding gestures, adolescents with higher schizotypy produced fewer gestures in a study.^25^

The processing and production of gestures can be investigated in multiple ways. On the one hand, simple self-report questionnaires such as the Brief Assessment of Gesture (BAG^26^) scale can be used. On the other hand, responses to natural behavior can be analyzed.^27^ To assess the perception and understanding of speech accompanied by gestures, a gesture-speech matching task has been developed.^5,11,12,28,29^

There is evidence supporting the validity of self-reported measures of gesture perception and production. Scores on the BAG scale were related to individual differences in empathy, highlighting its social relevance.^26^ Furthermore, neural activity during the speech-gesture matching task was correlated with self-reports about gesture sensitivity.^29^ However, it is unknown whether and how these measures are related to subdimensions of schizotypy.

Our first research question was: are schizotypal traits in a nonclinical sample related to gesture processing deficits? We expected that high values of overall schizotypy are related to reduced speech-gesture matching performance and self-reported gesture perception. We considered overall schizotypy to reduce the number of comparisons and facilitate comparison with previous studies using it.

Second, we wanted to explore how subdimensions of schizotypy are connected to self-reported and task-based measures of gesture processing. To study their complex multivariate relationships, we applied network modeling, an approach that allows extracting potential causal pathways in a data-driven manner.^30^ We computed bridge centrality and controllability values of nodes to infer which aspects of schizotypy and gesture processing are responsible for the association between these domains. This approach additionally allows for hypothesis testing. Based on findings in formal thought disorder in schizophrenia,^31,32^ we expected disorganized traits to be related to metaphoric gesture processing,^11^ and interpersonal schizotypy to be associated with impoverished self-reported production and perception of gestures in social situations. Regarding positive schizotypy, we expected a positive edge with appraisal of gestures made by others, but negative edges with speech-gesture matching, given its associations with hypermentalizing^33,34^ and blurred self-other discrimination.^35^

## Methods

### Participants

Participants were invited to enroll in the study through an online platform (https://www.soscisurvey.de). The online questionnaire was in German and was advertised widely amongst numerous mailing lists from the Universities of Marburg, Gießen, and Frankfurt and online forums across Germany. Overall 1379 subjects participated from the 9th of March 2019 until the 11th of May 2020. 1139 of them completed the task and the questionnaires of the survey. No exclusion criteria were applied. Only in case a test video (described in “Instruments”) could not adequately be described, we assumed technical problems or that a participant paid too little attention and did not consider the data from the gesture task in the analyses. This was the case in 4% of the participants leading to a final sample of 1094 datasets (mean age: 25.46, *SD* = 8.51, range: 17–80; sex: 69.65% female, 29.80% male, 0.55% divers). 92.47% of the participants reported to be healthy (reported no disorder/diagnosis) while 6.15% reported a mental disorder (psychiatric diagnosis; eg, depression or anxiety disorders), 0.09% reported a somatic diagnosis, and 1.29% a neurological diagnosis (eg, epilepsy).

### Instruments

**Fig. 1.**
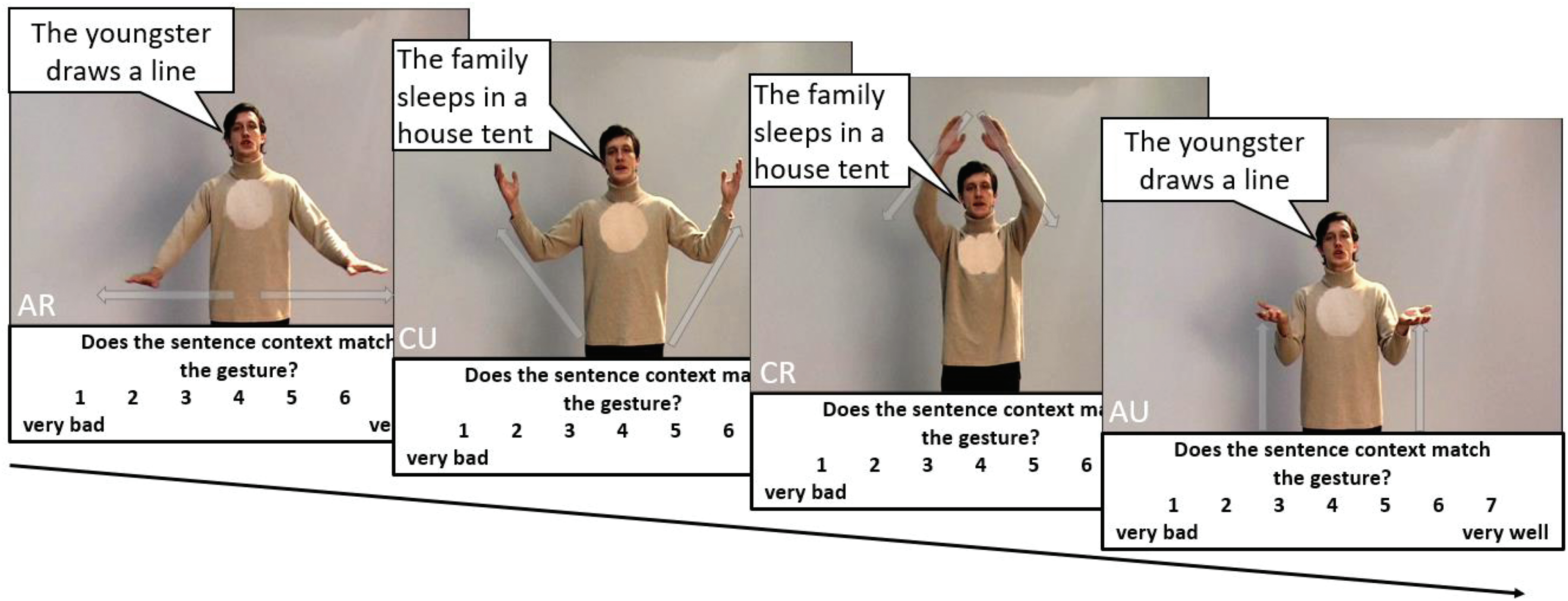
Short video clips of an actor reciting abstract (A, “draws a line”) or concrete (C, “house tent”) sentences accompanied by either a semantically related (R) or unrelated (U) gesture. Participants indicated on a scale from 1 to 7 whether they perceived each gesture as rather matching the speech content very well (7) or very bad (1). Eight videos were presented during the online study (2 × AR, 2 × AU, 2 × CR, 2 × CU). For a comparable procedure see refs. ^12,28^.

Participants were presented with 8 short video clips (5 s each) of an actor reciting abstract (A, metaphoric) or concrete (C, iconic) sentences accompanied by either a semantically related (R) or unrelated (U) gesture (see figure 1). For example, the concrete sentence “The ball is round” is accompanied by an iconic gesture illustrating the round shape of the ball. An abstract sentence could refer to the hierarchical structure of a company accompanied by a gesture illustrating the hierarchy levels with a metaphorical gesture. For each video, participants need to evaluate the alignment between speech and gesture on a 7-point Likert scale (1: low relatedness to 7: high relatedness; see refs. ^12,28^). We ensured that participants paid attention and could hear the sound properly with a test video that we included right before the speech-gesture matching task. We asked the participants if they could hear the spoken sentence of the test video and asked them to write down the content of the spoken sentences.

Furthermore, the BAG scale was applied as a self-report measure of gesture perception and production.^26^ The BAG scale consists of 12 items (1–5 agreement rating) and can be divided into 4 subscales: (1) perception (eg, “During a lecture, it’s very distracting to me if the speaker gestures a lot.”), (2) production (eg, “I’ve been told before that I gesture a lot when I talk”), (3) social perception (eg, “When I see someone gesturing a lot, I often wonder if I would have used the same gestures”), and (4) social production (eg, “When talking in noisy places, I usually gesture a lot to make myself understood over the noise”). BAG assessments had been related to multisensory processing,^36^ to neural mismatch responses of the bilateral inferior frontal gyrus,^29^ and potential impairments in patients with major depression.^37^ The internal consistency (Cronbach’s alpha) of the 4 subscales yielded good to acceptable results. The subscale internal consistency ranged between 0.74 (for gesture perception) and 0.49 (for social gesture perception^26^).

Schizotypal traits were measured using the self-report scale Schizotypal Personality Questionnaire—Brief 22-item version (SPQ-B),^38^ as also used in recent large ENIGMA initiatives.^39^ Based on the SPQ-B, one can compute a total scale score and 3 subscale scores that parallel the main symptom domains of schizotypal personality disorder: cognitive-perceptual alterations, interpersonal deficits, and disorganization.

### Statistical Analysis

First, we explored the bivariate relationships between general schizotypal traits and gesture performance in the speech-gesture matching task and gesture self-ratings using Spearman’s correlations. Then, to infer the network structure of schizotypal personality traits and objective and subjective aspects of gesture processing, we entered the subscale scores of the SPQ-B and the BAG, and performance summary measures for the objective gesture processing tasks into a network model. We estimated a non-regularized partial Spearman correlation network with the GGMnonreg package.^40^ We favored this approach as recent work has indicated that it is preferable when the number of observations greatly exceeds the number of variables.^41^ We used a nonparametric bootstrap approach implemented in the package, which applies a decision rule for edge inclusion that relies on percentile-based confidence intervals (95%) of edge weights.^41^ We used 5000 bootstrap samples, and the alpha was set to 0.05.

We used the estimated network structure to infer the relationships between the constructs and their importance. Furthermore, we wanted to determine which nodes are forming the links between 3 domains: (1) objective gesture processing, (2) subjective gesture processing and production, and (3) schizotypal personality traits. First, we performed community detection with the fast greedy algorithm^42^ to establish that the theoretically expected domains can be extracted in a purely data-driven way. In the network science literature, community detection refers to techniques that can detect sets of nodes that are more strongly connected to each other, as compared with the rest of the network. In the context of psychological networks, community detection can be applied as a dimension-reduction tool.^43,44^ We chose the fast greedy algorithm because it has no tuning parameter (reducing degrees of freedom in the analysis, thereby reducing potential researcher bias) and it is deterministic (facilitating reproducibility), and also because it tends to return large communities (facilitating interpretation).^45^ Simulation studies demonstrated it performs well in terms of recovering dimensions of the data.^43^ We also established whether the obtained communities were theoretically meaningful by comparing them against our theoretical expectation of the 3 domains we outlined above. Then, we calculated bridge centrality scores with the *networktools* package to identify the narrower aspects of schizotypal personality, and self-reported and objective gesture processing that are points of overlap between said domains.^46^ More specifically, we calculated bridge expected influence (taking into account only the direct connections of a node with nodes in other communities), bridge closeness (taking into account average distance to nodes in different communities), and bridge betweenness (forming shortest paths across communities). In the current study, we calculate bridge centrality scores to identify nodes that might be responsible for the co-occurrence of gesture processing alterations and schizotypal personality traits (akin to comorbidity between disorders).^47^ We evaluated the significance of the differences with bootstrapping.

Furthermore, we calculated the average controllability and the modal controllability to estimate how changes in a given network node would change the whole network. Average controllability in a network quantifies a node’s ability to guide the network into a wide range of states, signifying its role in promoting system adaptability and diversity. Modal controllability assesses a node’s capacity to propel the network toward rare or specific states, highlighting its importance in triggering unique or less common network configurations. To implement these measures, we used customized MATLAB scripts.^48^

## Results

**Fig. 2.**
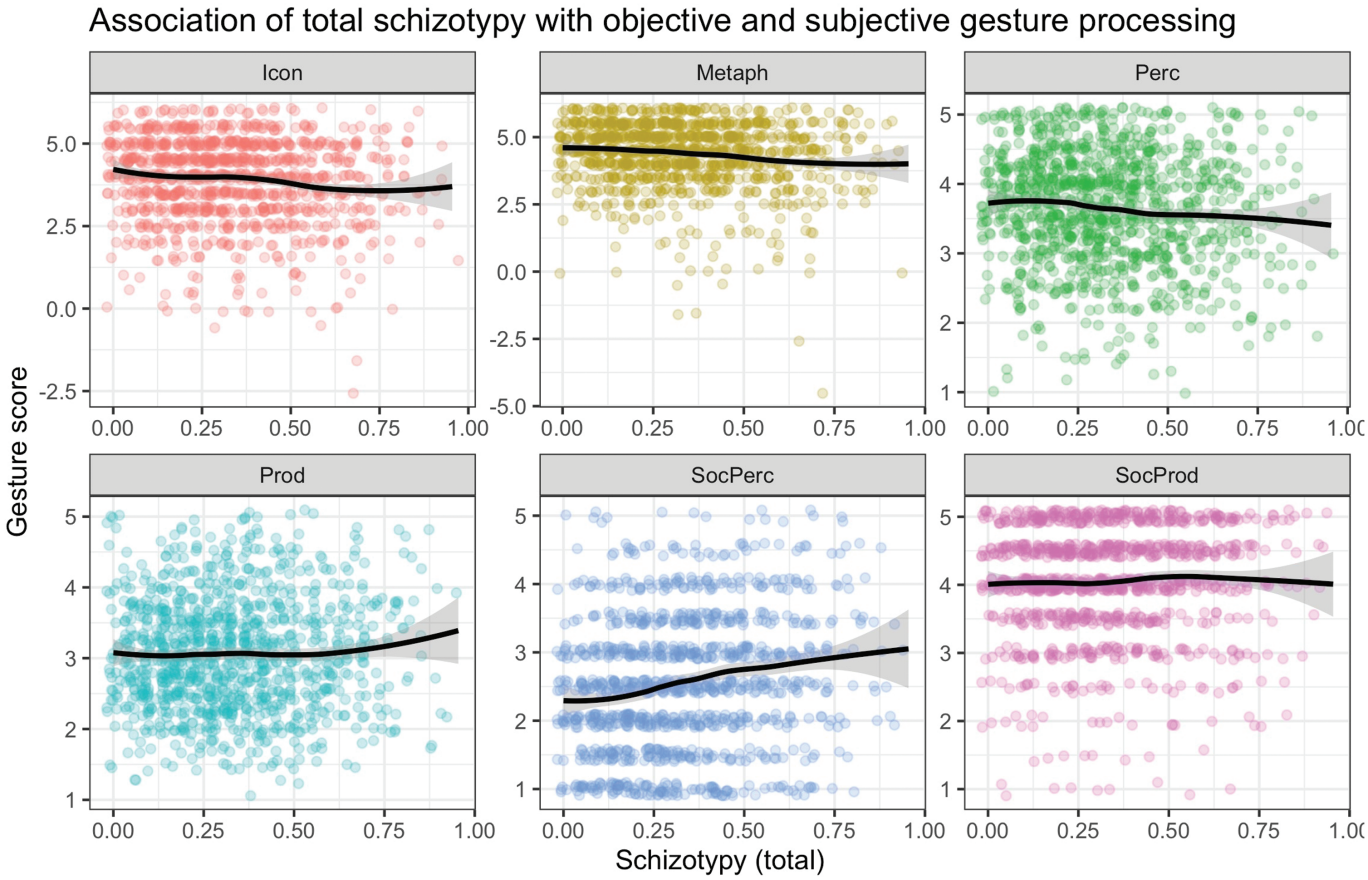
Bivariate association of total schizotypy with subjective and objective gesture processing. Locally estimated scatterplot smoothing (LOESS) trendlines are shown (with 95% confidence intervals shaded) for illustration purposes as these can capture nonlinear associations, aligned with the use of Spearman rank correlations for network estimation. A jitter was added to facilitate the visibility of individual data points. *Note*: BAG, Brief Assessment of Gesture; Icon, perception of iconic gestures; Metaph, perception of metaphoric gestures; Perc, BAG perception subscale; Prod, BAG production subscale; SocPerc, BAG social perception subscale; SocProd, BAG social production subscale.

**Fig. 3.**
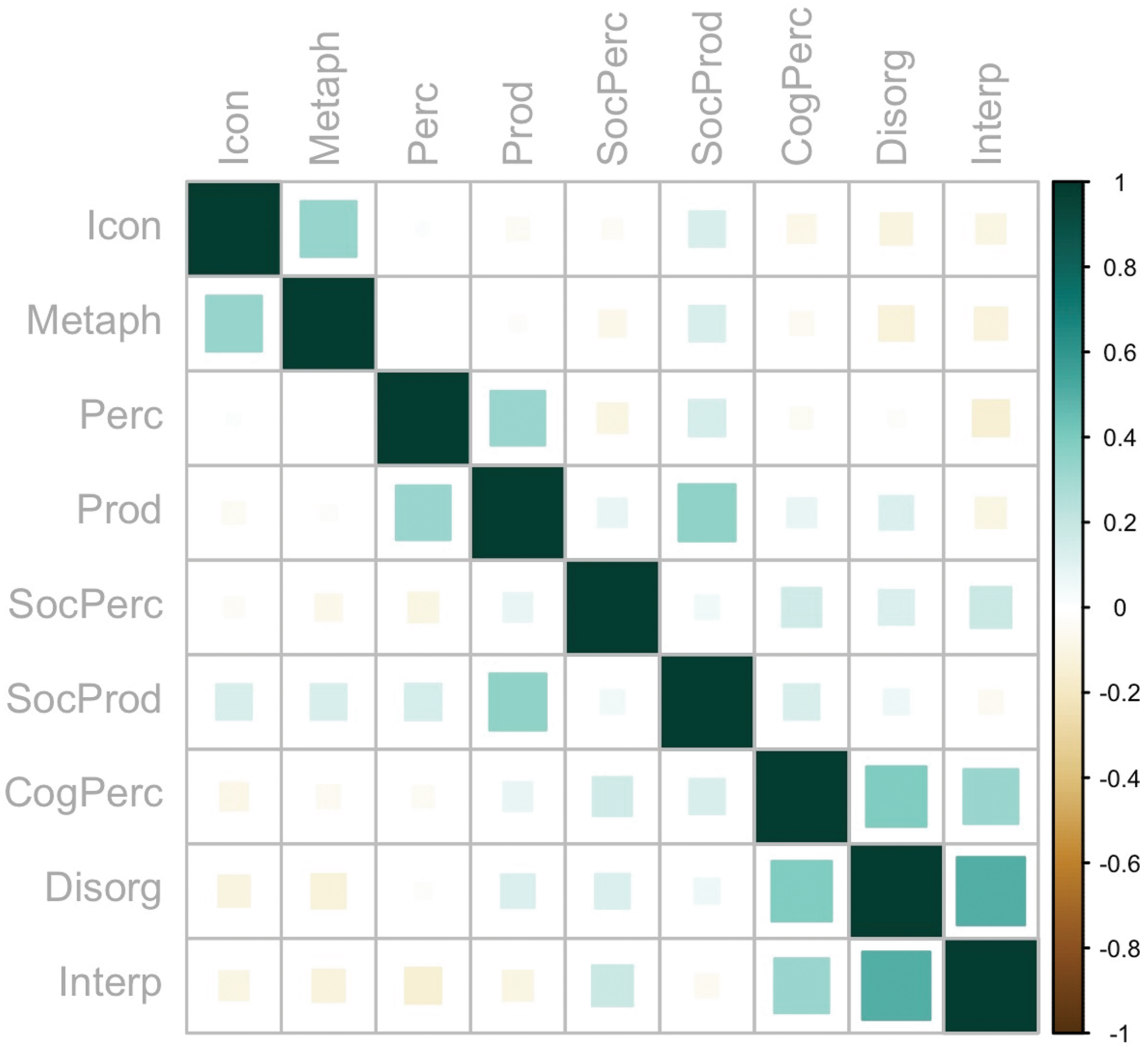
Spearman correlations between schizotypal personality traits, self-reported gesture processing and production, and gesture recognition performance. *Note*: BAG, Brief Assessment of Gesture; CogPerc, SPQ-B cognitive-perceptual subscale; Disorg, SPQ-B disorganization subscale; Icon, perception of iconic gestures; Interp, SPQ-B interpersonal subscale; Metaph, perception of metaphoric gestures; Perc, BAG perception subscale; Prod, BAG production subscale; SocPerc, BAG social perception subscale; SocProd, BAG social production subscale.

First, we tested the bivariate relationships between schizotypy and gesture performance and gesture self-ratings with Spearman correlations. We found negative correlations between schizotypy sum score and speech-gesture matching performance for concrete (*r* = −.114, *P* < .001) and abstract/metaphoric videos (*r* = −.122, *P* < .001), consistent with impairments found in schizophrenia.^5,11^ Furthermore, self-reports of gesture perception correlated negatively with schizotypal traits (*r* = −.102, *P* = .001). However, no correlations were found between overall schizotypy and gesture production (BAG Perception: *r* = .023, *P* = .448), social gesture production (BAG Social Production: *r* = .048, *P* = .112), and we even detected a positive correlation between overall schizotypy and social gesture perception (BAG Social Perception: *r* = .202, *P* < .001) (note that this subscale does not reflect social perception skills, but rather the appraisal of gestures, ie, whether someone tends to be amazed or surprised by others using gestures). For descriptive statistics and an overview illustration of all correlations between measures, see figure 2 and supplementary material. These inconsistent findings for the BAG subscales support the idea that a subscale-specific analysis in the context of a network approach is promising to reveal new insights into the subdomain associations. For descriptive purposes, we present the bivariate correlations in figure 3, and use network analyses to draw more specific conclusions.

**Fig. 4.**
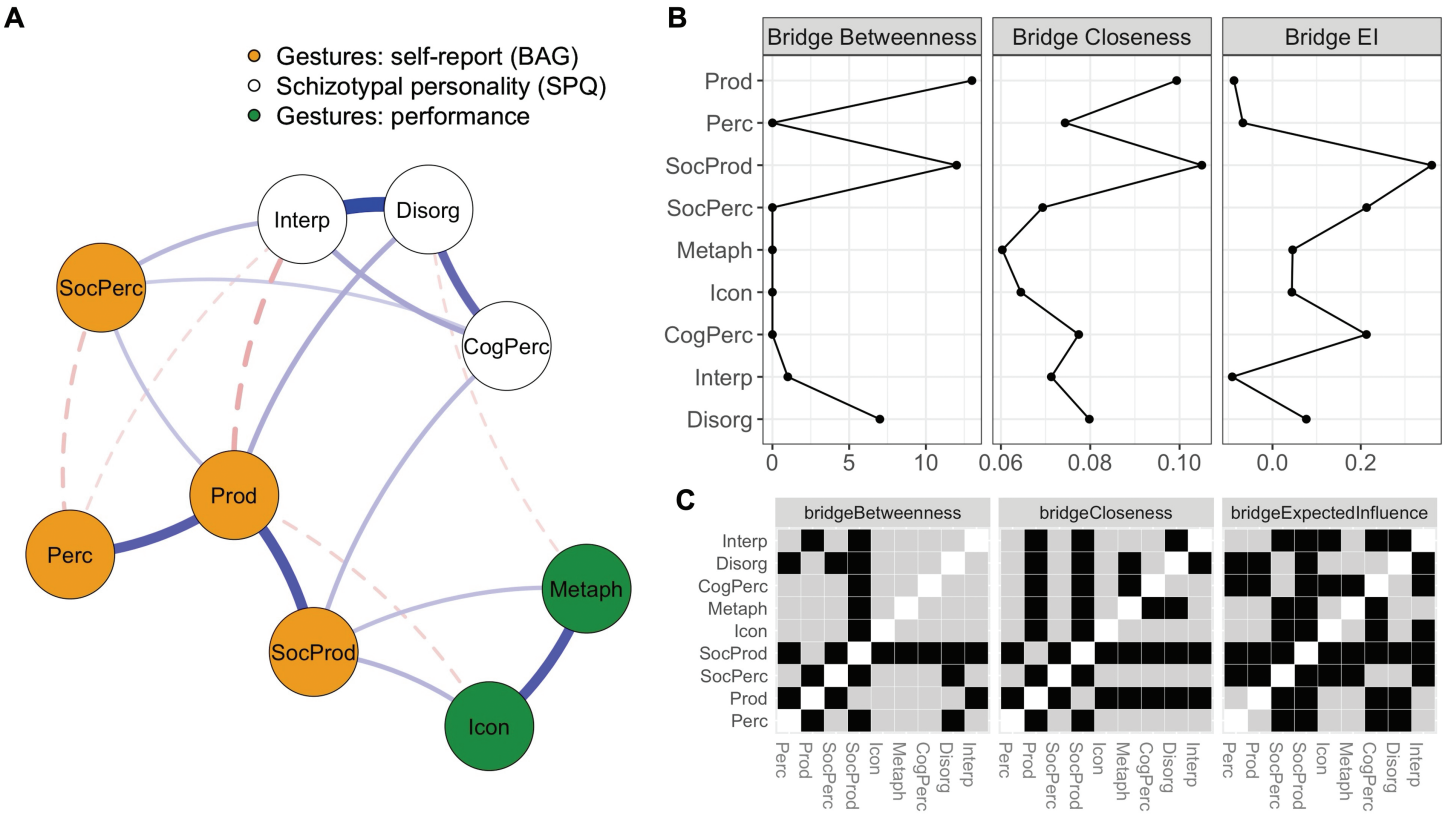
The network structure of schizotypy, gesture processing task performance, and self-reported gesture processing and production. (A) Estimated network structure and communities detected with the data-driven fast greedy algorithm. Blue, solid edges show positive and red, dashed lines show negative associations, respectively. Edge weight reflects the strength of the associations. (B) Bridge centralities based on the community structure shown in panel A. (C) The results of bootstrapped difference tests of bridge centrality metrics; black cells indicate significant differences (*P* < .05). *Note*: BAG, Brief Assessment of Gesture; Bridge EI, bridge expected influence; CogPerc, SPQ-B cognitive-perceptual subscale; Disorg, SPQ-B disorganization subscale; Icon, perception of iconic gestures; Interp, SPQ-B interpersonal subscale; Metaph, perception of metaphoric gestures; Perc, BAG perception subscale; Prod, BAG production subscale; SocPerc, BAG social perception subscale; SocProd, BAG social production subscale.

The estimated network is shown in figure 4A (see supplementary figure 1 for confidence intervals of edge weights). Schizotypy dimensions were all positively connected to each other, and disorganization showed strong links with the 2 other dimensions, particularly with interpersonal schizotypy traits. Cognitive-perceptual and interpersonal schizotypy were both positively related to self-reported social gesture perception, supporting our predictions regarding the altered appraisal of gestures being related to these subdimensions. In contrast, disorganized and interpersonal dimensions showed distinct and inverse associations with self-reported gesture production: interpersonal schizotypy was negatively, while disorganized schizotypy was positively linked. The former confirms our expectations, whilst, for the latter, we had no specific hypothesis. Reporting less efficient gesture perception was linked to interpersonal schizotypy, in line with our prediction. Self-reported social production, on the other hand, was only associated with cognitive-perceptual aspects of schizotypy. Performance on the iconic and metaphoric gesture processing tasks were strongly associated, and both were positively related to self-reported social gesture production. Yet, self-reported gesture production was negatively associated with iconic gesture processing. Finally, metaphoric gesture processing performance demonstrated a weak negative relationship with disorganized schizotypy, confirming our hypothesis, while our prediction regarding cognitive-perceptual schizotypy linked to disturbed speech-gesture processing was not supported.

We first established that the expected domains, corresponding to the 3 instruments, can be extracted in a purely data-driven way. The fast greedy community detection algorithm yielded 3 theoretically interpretable communities in the sense that they perfectly corresponded to the 3 instruments. Recall that communities are sets of nodes that are more interconnected with each other than with the nodes outside their community. In the context of the network approach to psychopathology, community detection can be used to estimate the number of dimensions.^44^ Here, the algorithm returned 3 communities corresponding to (1) self-reported gestures, (2) gesture recognition performance, and (3) schizotypal personality traits. Having established the content validity of the communities, then, we calculated bridge centralities to identify nodes that are important in connecting these domains, and tested the robustness of these differences with nonparametric bootstrapping (number of bootstrap replicates = 5000, figure 4C). We analyzed Bridge Betweenness, Bridge Closeness, and Bridge Expected influence (figure 4B). For each of them, BAG Soc Prod significantly stood out, while BAG Prod also stood out significantly in terms of Bridge Closeness. We can conclude that the self-reported production of gestures to enhance communication in social interactions is a factor that connects the domains of self-reported gesture production of perception, schizotypal traits, and gesture processing task performance. To assess to what extent the individual nodes are predicted by the rest of the network, we computed node predictability. This analysis is reported in supplementary materials.

Finally, we analyzed the network controllability. We found the highest average controllability for disorganized schizotypy (1.1241) followed by interpersonal schizotypy (1.1118) and gesture production (1.1028). All other values are in a similar range (BAG Social Production = 1.0586, cognitive-perceptual schizotypy = 1.0483, iconic gesture perception = 1.0464, metaphoric gesture perception = 1.0435, BAG Perception = 1.0421, BAG Social Perception = 1.0194). That indicates that changing something in gesture production or disorganized or interpersonal schizotypy would have a bigger impact on the whole network than changing one of the other notes.

## Discussion

Our aims were to explore the pattern of specific associations between schizotypy dimensions, and subjective and objective gesture processing, and to evaluate which aspects of schizotypy are more strongly associated with gesture processing and production. Another more global aim was to generate hypotheses about potential causal pathways for future intervention studies. At the level of pairwise associations, we established that people with heightened schizotypal traits have trouble matching gestures with speech. We observed this both for simple, concrete, and more difficult, abstract speech-gesture combinations, in concordance with results in patients with schizophrenia.^5,11,12^

In the network model, schizotypy dimensions were all positively connected to each other, and disorganization showed a particularly strong link with the 2 other dimensions, in line with previous studies.^49,50^ Performance on both gesture processing tasks was positively related to self-reported social gesture production, paralleling the association between impaired nonverbal social cognition with poor gesture performance in patients with schizophrenia.^51^ In contrast, self-reported gesture production was negatively related to the matching of iconic gestures with concrete speech. Interestingly, task performance was not directly connected to self-reported gesture perception. This suggests that the subjective aspect of gesture production, rather than perception, may better reflect one’s speech-gesture matching abilities.

We provided further evidence for why we should take a closer look at gestures^10^ as they are connected to specific dimensions of schizotypal traits. Disorganized and interpersonal dimensions showed inverse associations with self-reported gesture production: as expected, interpersonal schizotypy had a negative, while disorganized schizotypy, unexpectedly, had a positive link. Heightened self-reported production of gestures in social situations, on the other hand, was only associated with elevated cognitive-perceptual schizotypy, which was not predicted. Being amazed or surprised by others using gestures, as reflected by the social perception subscale, was linked to both interpersonal and cognitive-perceptual schizotypy. This is in line with our predictions and may reflect hypermentalizing.^34^ Confirming our predictions, there was a negative edge between disorganized schizotypy and metaphoric speech-gesture matching; this is in line with a specific impairment of metaphoric gesture processing in patients with formal thought disorders^11^ and more broadly, with the idea of an overlap between schizotypy in the general population and symptoms of schizophrenia.^15–17^ This finding might be specific to the multimodal speech-gesture domain as general metaphor processing seems to be intact in schizotypy.^52^ We expected direct edges between cognitive-perceptual schizotypy and speech-gesture matching but these were not observed.

The association of schizotypy with speech-gesture matching performance, however, seems to be also mediated by individual differences in self-reported gesture production. Multiple bridge centrality metrics revealed that self-reported social production of gestures was critical in connecting the domains of self-reported gesture production and perception, schizotypal traits, and speech-gesture matching performance. Importantly, this supports the validity of the BAG subscales for the investigation of subjective gesture preferences in a clinical and subclinical context: the BAG production scales connected schizotypal traits with an objective, performance-based measure of gesture processing. The tight connection between the gesture subscales and schizotypy subscales suggests that verbal and nonverbal communication are important aspects of mental health. The social production of gestures is also reduced in patients with major depression^37^ and thus might capture social impairments across disorders; this motivates the extension of the present framework to transdiagnostic clinical samples.

The network model facilitates the formulation of causal hypotheses. One could argue for 2 types of interventions on a network: changing the node’s “activity” and modifying edges. For controllability, we assume that the values of the nodes are adaptable. We found the highest average controllability for disorganized and interpersonal schizotypy, followed by self-reported gesture production. While schizotypy is conceptualized as a trait, ie, temporally stable by definition, changes in gesture production are probably easier to target with gesture training or brain stimulation.^28^ Based on our study, one may predict that in patients, improving speech-gesture matching by brain stimulation,^12,53^ potentially combined with psychotherapy,^54^ would primarily alleviate disorganization and benefit the social production of gestures. Furthermore, gesture training might not only change the neural mechanisms that implement gesture processing^55^ but could also modify the network structure more globally, which might explain how gesture training could improve the quality of life in patients with schizophrenia.^56^

Overall, it remains speculative whether, across the lifespan, schizotypal traits cause gesture alterations or vice versa. During childhood and adolescence, inefficient gesture processing can hinder interpersonal functioning, which may contribute to the development of schizoid personality traits.^18,19^ It is further possible that schizotypy and speech-gesture matching have shared neurodevelopmental roots. Longitudinal assessments of gesture, schizotypal traits, and structural and functional magnetic resonance imaging could clarify this issue. Additionally, intervention studies may test the causal hypotheses our study has generated.

More broadly, our study contributes to the discussion around the convergence between behaviorally measured and self-reported indicators of the same constructs. Recently, little correspondence was found between performance on tasks and questionnaire scores within the domains of self-regulation,^57^ impulsivity,^58^ and reward and punishment sensitivity,^59^ and self-reports outperformed behavioral indicators when it came to predicting real-life outcomes^57,59^ Our findings paint a less pessimistic picture: we found a correspondence between objective gesture processing performance and self-reported gesture production, and the latter, in turn, was associated with self-reported gesture processing and schizotypy. However, the lack of a direct connection between self-reported and objectively assessed gesture perception raises the possibility that individuals might still have poor self-evaluation in this regard. Notably, we also found a specific association between disorganized schizotypy and subtle impairments in understanding metaphoric gestures, thereby establishing the predictive validity of a behavioral measure.

The current investigation is not without limitations. First of all, the construct validity of the SPQ-B is different from other schizotypy instruments.^60^ Specifically, the interpersonal subscale of the SPQ-B does not cover classical negative schizotypy traits (physical and social anhedonia, and amotivation).^61^ The interpersonal scale showed an association with positive schizotypy in the present study, which contrasts with measures of classical negative schizotypy that do not correlate with positive schizotypy, after disorganization is partialled out.^49,50^ Conceptually, it is unlikely that physical anhedonia would be related to gesture processing. Social anhedonia might be indirectly related to gesture processing impairments: not being able to process subtle gestures may make social interactions less rewarding, or, someone who does not enjoy social interactions may get less exposure to opportunities for developing gesture processing. Future research should clarify how anhedonia and amotivation are related to gesture processing. Furthermore, gesture production abilities were not assessed objectively and self-reports might be distorted by metacognitive biases associated with schizotypy.^62^ A further limitation is the online context of the study where reports of the subjects cannot be validated by the experimenter. An infrequency scale was not included, which could have been used to screen careless responders. Additionally, in the present study, the speech-gesture task included only 8 Likert ratings (1–7), for the sake of brevity. These may increase random measurement error, which was compensated for by the large sample size. Furthermore, we aimed to increase engagement and improve data quality with an attention check and a sound check right before the speech-gesture matching task. In future studies, more nuanced and extensive measures might be considered, although there is an unavoidable trade-off between task length and sample size. As the sample is not balanced regarding sex and age, we need also to consider a potential bias in our sample with an overrepresentation of younger female participants. Sex differences in the association between schizotypy and (social) cognition are understudied, making it hard to evaluate how sex imbalance in the sample could confound the findings. Additionally, we can argue that the overrepresentation of females is only mild, as about 30% of the participants are male. Finally, we acknowledge that drawing causal conclusions with observational data relies on many assumptions (such as no unmeasured confounders and temporal precedence), which might not be met in the present study. Nevertheless, a valuable contribution of this study is the generation of causal hypotheses, which can later be verified experimentally.

In conclusion, here, we explored the link between understanding speech and gestures, and interpersonal, disorganized, and cognitive-perceptual schizotypy. Our findings provide new insights into how such a network might be efficiently changed as indicated by high controllability scores. This is especially important as potential interventions, such as noninvasive brain stimulation^12,53^ or gesture training^56^ are available and have been successfully applied to speech-gesture processing or general gesture performance in schizophrenia. However, the causal pathways will need to be clarified by clinical experimental studies. Furthermore, a careful cost-benefit analysis must inform a choice between brain stimulation and behavioral interventions (or combinations thereof) in patients, taking efficiency and undesirable effects into account. In general, our findings demonstrate the relevance of combining objective and subjective measures in a network to understand social-communicative functioning.

## Supplementary Material

Supplementary material is available at https://academic.oup.com/schizophreniabulletin/.

sbae134_suppl_Supplementary
