# Supplementary material for: Speech-Gesture Matching and Schizotypal Traits: A Network Approach
Source: Schizophr Bull. 2024 Jul 24;51(4):1009–18. doi: 10.1093/schbul/sbae134 (PMC12236348; doi:10.1093/schbul/sbae134)
Supplement: sbae134_suppl_Supplementary [file sbae134_suppl_supplementary.docx]

# Supplementary Materials - Speech-gesture matching and schizotypal traits: A network approach


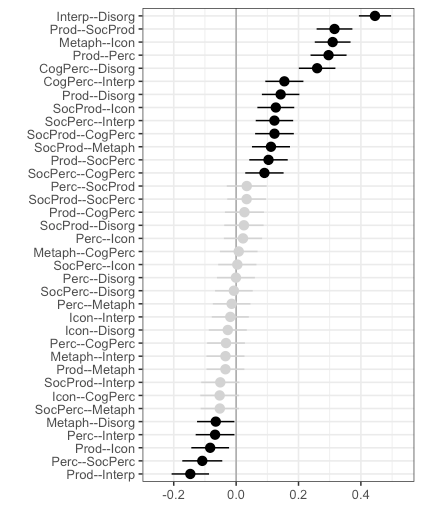


**Supplementary Figure 1.** Edge weight estimates with 95% confidence intervals. Significant edges (interval excludes zero) are shown in black, while nonsignificant edges ( interval includes 0) are shown in gray.
Icon: perception of iconic gestures; Metaph: perception of metaphoric gestures; Perc: BAG perception subscale; Prod: BAG production subscale; SocPerc: BAG social perception subscale; SocProd: BAG social production subscale; CogPerc: SPQ-B cognitive-perceptual subscale; Disorg: SPQ-B disorganization subscale; Interp: SPQ-B interpersonal subscale. Bridge EI: bridge expected influence.


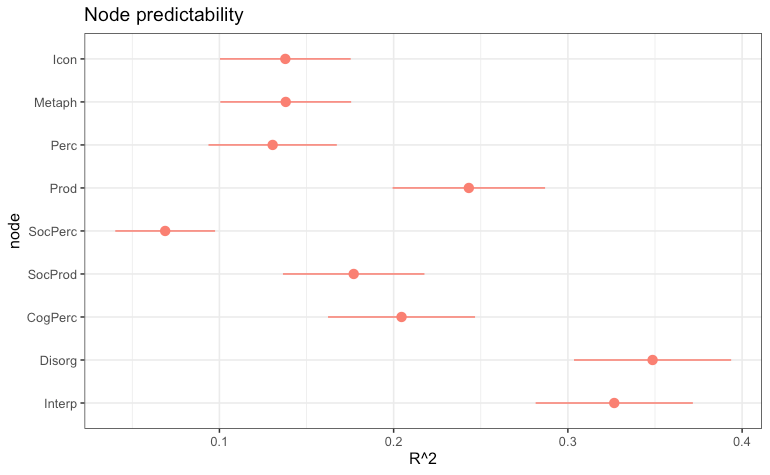


**Supplementary Figure 2. Node predictability values.** Error bars represent 95% confidence intervals.

Icon: perception of iconic gestures; Metaph: perception of metaphoric gestures; Perc: BAG perception subscale; Prod: BAG production subscale; SocPerc: BAG social perception subscale; SocProd: BAG social production subscale; CogPerc: SPQ-B cognitive-perceptual subscale; Disorg: SPQ-B disorganization subscale; Interp: SPQ-B interpersonal subscale.

We asked to what extent schizotypy subdimensions, subjective gesture production and processing, and gesture processing task performance are determined by each other. To answer this, we calculated node predictability, which gives an absolute metric of determination of a node by its neighbors. Nodes with higher predictability are more determined by the rest of the network. Bootstrapped confidence intervals were constructed for predictability estimates.

Node predictabilities are shown in Supplementary Figure 2. Notably, interpersonal and disorganized schizotypy were the most determined by the network, far above cognitive-perceptual schizotypy. This suggests that interpersonal difficulties and disorganized speech and behavior are more strongly associated with gesture processing and schizotypy, as compared to unusual beliefs, suspiciousness, and anomalous perceptions (note that the strongest edge in the network was between interpersonal and disorganized schizotypy, which may partially explain the high predictability). Within the set of indicators of gesture processing and production, self-reported production had the highest while social perception had the lowest predictability. Thus, perception of social gestures might be related to factors that are not modeled in the network. Self-reported perception and social production, and objectively measured iconic and metaphoric gesture production had intermediate predictability within this set.


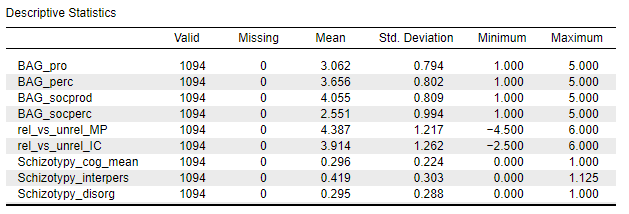


**Supplementary Table 1. Descriptive statistics.**
